# Nox, Reactive Oxygen Species and Regulation of Vascular Cell Fate

**DOI:** 10.3390/antiox6040090

**Published:** 2017-11-14

**Authors:** Denise Burtenshaw, Roya Hakimjavadi, Eileen M. Redmond, Paul A. Cahill

**Affiliations:** 1 Vascular Biology & Therapeutics, School of Biotechnology, Dublin City University, D09NR58 Dublin, Ireland; denise.burtenshaw3@mail.dcu.ie (D.B.); roya.hakimjavadi@dcu.ie (R.H.); 2 Department of Surgery (Research), University of Rochester Medical Centre, Rochester, NY 14620, USA; eileen_redmond@urmc.rochester.edu

**Keywords:** Nox, ROS, vascular smooth muscle, endothelial, stem cells, adventitial cells, arteriosclerotic disease, intimal-medial thickening

## Abstract

The generation of reactive oxygen species (ROS) and an imbalance of antioxidant defence mechanisms can result in oxidative stress. Several pro-atherogenic stimuli that promote intimal-medial thickening (IMT) and early arteriosclerotic disease progression share oxidative stress as a common regulatory pathway dictating vascular cell fate. The major source of ROS generated within the vascular system is the nicotinamide adenine dinucleotide phosphate (NADPH) oxidase family of enzymes (Nox), of which seven members have been characterized. The Nox family are critical determinants of the redox state within the vessel wall that dictate, in part the pathophysiology of several vascular phenotypes. This review highlights the putative role of ROS in controlling vascular fate by promoting endothelial dysfunction, altering vascular smooth muscle phenotype and dictating resident vascular stem cell fate, all of which contribute to intimal medial thickening and vascular disease progression.

## 1. Introduction

The major reactive oxygen species (ROS)-producing systems within the vascular bed include the reduced form of nicotinamide adenine dinucleotide phosphate (NADPH) oxidase [1], xanthine oxidase [2], the mitochondrial electron transport chain [3], and uncoupled endothelial nitric oxide (NO) synthase [4]. The Nox family of NADPH oxidases are an important source of ROS and are critical determinants of the redox state of the vessel wall. Changes in the expression of Nox can dictate, in part, the pathophysiology of several vascular phenotypes since ROS production may exceed the available antioxidant defence systems, thereby facilitating oxidative stress [5]. 

ROS can be either dysfunctional or protective. Superoxide anions (O_2_^−^) react with NO to form peroxynitrite (ONOO^−^) that causes nitric oxide synthase (NOS) uncoupling in endothelial cells (EC) and limits the amount of NO that is protective. Peroxynitrite (ONOO^−^) also induces thiol oxidation as well as tyrosine nitration causing further damage to the vasculature [6]. Moreover, the dismutation product of O_2_^−^, hydrogen peroxide (H_2_O_2_), directly promotes vascular smooth muscle cell (vSMC) hypertrophy, activates metalloproteinases, and at higher concentrations, inhibits endothelial NOS (eNOS) by phosphorylation of tyrosine 657 through the redox-activated tyrosine kinase Pyk2 [7]. H_2_O_2_ can also activate protein kinase-G Iα to induce thiol oxidation and subsequent dimerization [8]. In some instances, H_2_O_2_ may be protective and activate eNOS to counteract the pro-atherogenic effects of various pathological stimuli [8].

Arteriosclerosis is an important age-dependent condition that includes atherosclerosis, pulmonary hypertension, in-stent restenosis, autologous bypass grafting and transplant arteriosclerosis and may result in a heart attack or stroke [9]. A hallmark of arteriosclerosis is a pathologic vascular fibrosis due to the accumulation of vSMC-like neointimal cells resulting in intimal-medial thickening (IMT) and lesion formation that significantly narrows the vessel lumen but also provides a substrate for lipoprotein retention leading to accelerated atherosclerosis [9]. 

Animal studies have provided compelling evidence supporting a role for oxidative stress in contributing to IMT and the progression of early arteriosclerotic disease [6]. Since vascular lesions develop preferentially within regions exposed to disturbed blood flow patterns resulting in enhanced oxidative stress, the role of ROS in controlling endothelial dysfunction during the initiation of vascular pathologies has attracted extensive interest [10]. Subsequently, the important modulatory role of ROS in controlling de-differentiation and reprogramming of vSMCs [11] in addition to myogenic differentiation of resident vascular stem cell niche(s) and transforming growth factor β1 (TGF-β1) induced endothelial-mesenchymal stem cell transition (EndoMT) has emerged [12,13,14].

Stem cells play an important role during developmental stages due to their unique ability to self-renew and differentiate. However, their putative role in vascular disease progression has only recently attracted considerable interest [15]. Many types of stem cells can be cultivated in vitro including embryonic (ESCs), pluripotent (iPSCs), and mesenchymal (MSCs), each with the ability to differentiate into vascular cell lineages via progenitor cells [16]. In the past decade, numerous studies have demonstrated myogenic differentiation of mesenchymal-like stem cells to vSMC lineages in vitro following appropriate inductive stimulation [17], and in vivo, using transgenic Cre-LoxP marked cells after iatrogenic injury in murine models of IMT [12,18,19]. However, little is known about the metabolic changes occurring during the myogenic differentiation process [20]. Several studies have demonstrated the importance of the regulation of redox states by Nox isoforms in stem cell maintenance, proliferation and differentiation [21]. 

Cardiovascular risk factors such as hypercholesterolemia, hypertension, diabetes mellitus and smoking all enhance ROS generation and exacerbate the decrease in endothelial NO production [6]. Moreover, key molecular events during vascular disease progression such as oxidative modification of lipoproteins and phospholipids [22], EC activation and permeability changes [23] and disruption of the glycocalyx [24] and cellular infiltration/activation are facilitated by vascular oxidative stress [5].

It is commonly recognised that high levels of ROS may have destructive effects on both differentiated vSMC and EC [25,26]. This review will discuss the importance of Nox generation of ROS in controlling EC, vSMC and mesenchymal-like stem cell function and fate and their contribution to IMT and vascular disease progression.

## 2. Vascular Nox/Duox Isoforms

Low to moderate levels of ROS are known to contribute to important functions such as cell differentiation, migration, adhesion, senescence, growth, and apoptosis. In contrast, a variety of cardiovascular diseases are associated with elevated ROS levels. A significant source of cellular ROS is the Nox family of enzymes [27]. Initially, the over production of ROS such as O_2_^−^, H_2_O_2_, NO, hydroxyl radicals, ONOO^−^ and other metabolites within a cell system was labelled as a random event [28]. However, in recent years, studies have confirmed that ROS is a controlled signalling system with enzymes Nox and dual oxidase (Duox) at the forefront [29,30].

**Figure 1:**
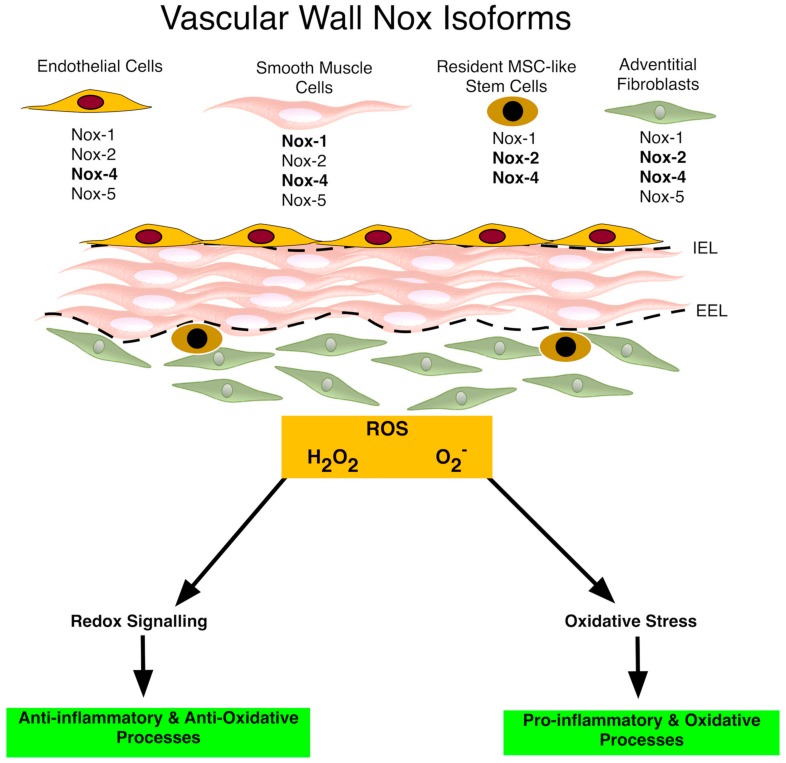
Nox enzymes within the vasculature. The schematic depicts the Nox enzymes within the vascular wall. All three layers of the vascular wall [intima (i.e., endothelial cells), media (i.e., smooth muscle cells), and adventitia (i.e., fibroblasts and macrophages)] express Nox family members. Nox4 is the predominant isoform in endothelial cells, Nox1 and Nox4 in smooth muscle cells, Nox4 in fibroblasts, and Nox2 and Nox4 in stem cells. Nox-derived O_2_^−^ avidly reacts with NO. Nox-derived ROS also affect the extracellular matrix and the external (EEL) and internal elastic lamina (IEL), influence gene expression and are involved in cell proliferation, migration and differentiation that lead to vascular disease progression [55].

In general, mammals have seven isoforms of Nox enzymes; Nox1, 2, 3, 4 and 5, Duox1 and Duox2. The seven classified Nox proteins are present not only in the plasma membrane, (Nox1 to 5 and Duox1/2), but also in the endoplasmic reticulum, (Nox2, 4 and 5), the mitochondrial membrane (Nox4) and the nuclear membrane, (Nox4 and 5) [31]. Within the vasculature, Nox isoforms are found in ECs [32], vSMCs [33], adventitial fibroblasts [34] and stem cells [35] (Figure 1).

All Nox enzymes are heteroprotein transmembrane complexes (except Nox5) with specific regulatory mechanisms, tissue specificity and downstream targets and functions. They contain six transmembrane domains with conserved binding sites for flavin adenine dinucleotide (FAD) and NADPH and four haem-binding histidines in the third and fifth transmembrane domains to produce ROS by transferring an electron to molecular oxygen [36]. Each member of the Nox family retains the catalytic domain allowing the transfer of electrons from cytosolic NADPH during the generation of ROS metabolites. This transfer leads to the reduction of oxygen by Nox/Duox to O_2_^−^ as well as to secondary ROS by-products [37]. During this process, two O_2_^−^ molecules have the potential to react generating H_2_O_2_, either spontaneously, or driven by the enzyme superoxide dismutase (SOD) in a process called dismutation. H_2_O_2_ can further react with O_2_^−^ in the presence of iron, generating a hydroxyl radical. In addition, other ROS products can be generated including the production of hypochlorous acid from the reaction of H_2_O_2_ and chloride driven by the presence of the enzyme myeloperoxidase or the production of ONOO^−^ generated by the reaction of O_2_^−^ with NO [38]. The resulting bi-products of these chain reactions (H_2_O_2_, NO) possess beneficial functions in microbial killing, cell signalling, differentiation and gene regulation. However, other ROS products including hydroxyl radicals, hypochlorous acid and ONOO^−^ are more often associated with cellular damage and molecular pathologies in extreme conditions of oxidative stress [38]. 

The primary ROS product of Nox1, 2 , 3 and 5 is O_2_^−^; however, Nox4, Duox1 and Duox2 produce mainly H_2_O_2_ [39]. Despite similarities in core structures, Nox homologues have different mechanisms of activation. Nox2 is the most clearly defined [40]. On activation, p47phox is phosphorylated and translocates to the membrane by forming a complex with p67phox and p40phox. Phosphorylation of p47phox induces a conformational change in a tandem Src homology 3 (SH3) domain that facilitates binding to a proline-rich region in the cytosolic C-terminus of the transmembrane subunit p22phox [41,42]. Independently, the guanosine-5′-triphosphate GTP-binding protein Ras-related C3 botulinum toxin substrate (Rac) also moves to the membrane to enable activation. Nox1 also requires association with cytosolic components (p47phox, p67phox or NoxO1 and NoxA1). In contrast, the Nox4-based oxidase appears to be constitutively active and does not require p47phox, p67phox, or Rac. Recent studies suggest that Poldip2, a polymerase (DNA-directed) delta interacting protein-2 is a novel Nox4/p22phox interacting protein that positively regulates Nox4 [43]. The Nox4/p22phox/Poldip2 complex has profound effects on Rho-dependent cytoskeletal reorganization and cellular migration and may be fundamental to the physiological and pathophysiological role of Nox4 [43]. Duox, Duox2 and Nox5 require a calcium ion binding through the presence of a helix-loop-helix structural domain (EF-hand) [31] and are activated by an elevation in intracellular Ca^2+^ [44,45]. Once bound to the required membrane proteins, the activated Nox/Duox enzyme can successfully transfer electrons across the membrane [46]. 

In addition to being highly reactive and oxidizing proteins, DNA and lipids, ROS are now considered important signalling molecules in their own right, modulating cellular processes such as gene expression, proliferation, and migration. Importantly, Nox enzymes are the only enzymes whose primary function is to generate ROS [36]. Other enzymes (cyclooxygenases, cytochrome P450, enzymes of the mitochondrial electron transport chain) can produce ROS, but only as a by-product of their normal function. In addition, secondary sources of ROS are generated from “uncoupled” eNOS and xanthine oxidase, which are dysfunctional variants of eNOS and xanthine dehydrogenase, respectively [36]. 

Numerous stimuli promote sustained activation of vascular Nox including several growth factors (platelet-derived growth factor (PDGF) [47], epidermal growth factor (EGF) [48], and TGF-β1 [49]), cytokines [50], mechanical forces (cyclic stretch, laminar, and oscillatory shear stress) [51], metabolic factors (hyperglycemia, hyperinsulinemia, free fatty acids, and advanced glycation end products) [52], and G protein–coupled receptor agonists (serotonin, thrombin, bradykinin, endothelin, and angiotensin II [Ang II]) [53]. Signalling proteins, c-Src, p21Ras, protein kinase C (PKC), phospholipase D and phospholipase A_2_ (PLA_2_) all play key roles in vascular NADPH oxidase activation [54]. Multiple pathways of Nox activation have been described in various cell types under normal and pathological states including phosphorylation of cytosolic regulatory subunits by protein kinase C (PKC), protein kinase A (PKA), phosphatidylinositol-3-kinase (PI3K), mitogen-activated protein kinases (MAPK), and non-receptor associated protein kinases (e.g., Janus kinase (JAK) and the non-receptor tyrosine kinases, Src) [55]. Transient changes in intracellular ion fluxes and protein-protein interactions with members of the thioredoxin family may also promote Nox activity [55].

Redox regulation of vascular Nox activation provides important positive and negative feedback regulatory mechanisms [56]. Cellular redox status is maintained by intracellular redox-regulating molecules, including thioredoxin (TRX). Nuclear factor erythroid 2–related factor 2 (Nrf2), an antioxidant transcriptional factor is regulated by the intracellular redox state. Binding sites for Nrf2 are located in the promoter region of a large variety of genes, including Nox4 [55]. These self-limiting negative feedback mechanisms predominate during physiologic conditions but may also be involved in maintaining low output of the nonphagocyte Nox, whereas a positive mechanism may predominate in a variety of vascular diseases [36].

## 3. Nox and Vascular Endothelial Cells

The role of Nox isoforms within the intimal endothelial layer has been the subject of several studies. The vascular endothelium plays a critical role in cardiovascular homeostasis. Vascular endothelial cells normally perform key homeostatic functions such as maintaining blood fluid, regulating blood flow, regulating macromolecule and fluid exchange with the tissues and preventing leukocyte activation [57]. Endothelial dysfunction is characterized by a shift in the actions of the endothelial cell towards a reduced endothelium-dependent vasodilation (via reduced bioavailability of NO and prostacyclin) and promotion of inflammatory and prothrombotic state [57]. These changes are associated with most forms of cardiovascular disease, such as arteriosclerosis, hypertension, peripheral vascular disease and diabetes. Alteration in endothelial function precedes the development of morphological changes in arteriosclerotic disease and contributes to IMT development and later clinical complications [57]. Low levels of Nox activity are evident under normal physiological conditions. Upregulation of the various Nox isoforms is associated with vascular pathology in response to injury underlying arteriosclerosis, diabetes, obesity, hypertension, and hypoxia [55]. In this regard, ECs express four Nox isoforms including the superoxide-generating enzymes Nox1, 2, and 5 and the hydrogen peroxide-generating enzyme Nox4 [36]. Under physiological conditions these enzymes are expressed at relatively low levels on membranes of the endoplasmic reticulum (ER) and nucleus where they contribute to intracellular redox signalling processes. However, in cardiovascular disease such as atherosclerosis, endothelial expression of Nox2, and to a lesser extent of Nox1, appears to be enhanced, resulting in excessive superoxide generation—especially in the extracellular compartment [36].

**Figure 2:**
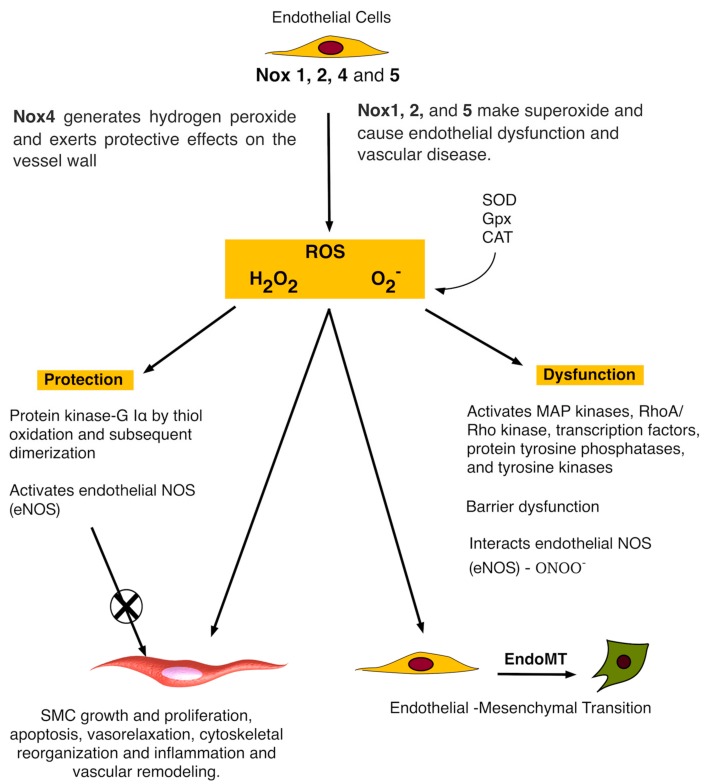
Simplified schematic of common forms of Nox enzymes in endothelial cells. H_2_O_2_ can be converted to H_2_O by catalase (CAT) or glutathione peroxidase (GPx). O_2_^−^ can also react with NO to generate the reactive nitrogen species ONOO^−^ that could be converted into NO_2_·, which reacts with protein tyrosine residues to generate NO_2_-Tyr. This reaction can lead to a decrease in the bioavailability of NO, leading to endothelial dysfunction. Nox-derived ROS may also control endothelial-mesenchymal stem cell transition (EndoMT) [36].

Recent studies on human arteries from patients with coronary artery disease (CAD) [58], and experimentally induced hypertension [40], diabetes and atherosclerosis [59] in animal model systems suggest that Nox1, 2 and 5 all promote endothelial dysfunction, inflammation, and apoptosis within the vessel wall [1], whereas Nox4 is by contrast vasoprotective via increasing NO bioavailability and suppressing apoptotic pathways (Figure 2). It is clear from most in vitro studies that the functional role of Nox4 is beneficial [60], in particular, by mediating an endothelium-derived hyperpolarizing factor (EDHF)-type endothelium-dependent vasodilatation to systemically lower blood pressure [61].

Endothelial-specific overexpression of Nox2 generates O_2_^−^ and promotes vascular dysfunction and oxidative stress [40]. Similar effects are reported following ectopic expression of Nox1 within vSMCs [62]. Hence, it is thought that Nox-derived O_2_^−^ reacts with endothelium-derived NO to lower its bioavailability and increase the generation of ONOO^−^. The free radical ONOO^−^ is highly reactive and oxidizes cysteines and tetrahydrobiopterin, an essential cofactor of the eNOS. Lack of tetrahydrobiopterin uncouples the enzyme, and promotes further O_2_^−^ generation exacerbating the dysfunction [63]. Unlike O_2_^−^, Nox4 derived H_2_O_2_ doesn’t react with NO but may induce NO synthase [64]. 

It is also clear that type of ROS and the site of production is of particular importance. Despite generating H_2_O_2_, ectopic expression of endothelial Nox4 did not promote medial hypertrophy in contrast to ectopic expression of Nox4 in vSMC or direct application of H_2_O_2_ [61]. The H_2_O_2_ may counteract the hypertrophy or react with peroxiredoxins, which subsequently mediate signalling by thiol-based redox reactions [65].

Although preclinical studies have provided compelling evidence for selective targeting of Nox1 and Nox2 in ECs, it remains unclear whether chronic inhibition of Nox activity in humans will also be efficacious. One inhibitor, apocynin, is thought to inhibit Nox activity by blocking phosphorylation of p47phox, and consequently its interaction with membrane-bound oxidase components [66]. Short-term administration of apocynin to humans is well tolerated and effective at reducing ROS production, but it is unclear whether the pharmacodynamic and pharmacokinetic profiles of apocynin are suited to long-term administration [67].

## 4. Nox and Vascular Smooth Muscle Cells

**Figure 3:**
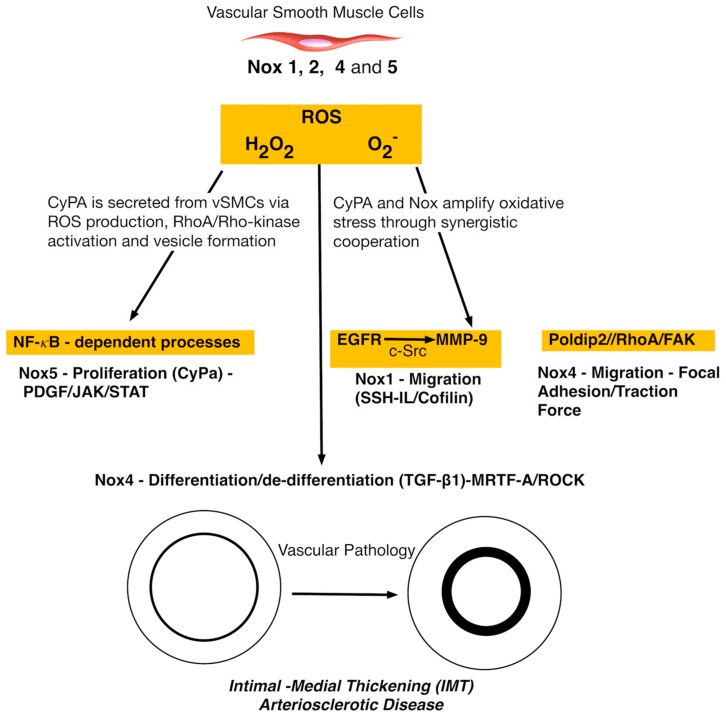
Simplified schematic of common forms of Nox enzymes in vascular smooth muscle cell (vSMCs). Nox4, once activated, generates H_2_O_2_. Nox1 expression is increased early and decreased with lesion progression, while induction of Nox4 is a late event. Nox2 and p22phox are elevated throughout lesion development. SMCs have increased generation of ROS, cell cycle arrest, evidence of senescence, and increased susceptibility to apoptosis [69].

The role of Nox isoforms within the medial layer has also been addressed. A hallmark of arteriosclerotic disease is the accumulation of vSMC-like cells resulting in IMT [9]. Several studies have focussed on the putative roles of different Nox isoforms in vSMC that generate ROS and the subsequent repercussions on vSMC cell phenotype and fate that contribute to vascular disease progression (Figure 3). The intracellular signalling pathways responsible for ROS generation are still unclear, but it is known that Nox1, 2, 4, and 5 are all expressed in vSMC but may differ in their activity, response to stimuli, and the type of ROS released [68].

Several recent studies have provided exciting new insight into the molecular mechanisms by which agonists, through Nox1 generation of ROS, may promote vascular lesion formation. Vascular SMC migration is pivotal to lesion formation and IMT and is considered an integrated, dynamic, and cyclical process dependent upon orchestrated control of regulators of the actin cytoskeleton, including cofilin. Cofilin is a major effector of Nox1-mediated vSMC migration in vitro and is notably reduced in Nox1-deficient (Nox1^−/−^) cells under both basal and PDGF-stimulated conditions [70]. Thrombin-induced shedding of N-cadherin and vSMC migration is mediated by Nox1-dependent transactivation of the epidermal growth factor receptor (EGFR) and subsequent activation of matrix metalloproteinase-9 (MMP-9). Nox1-dependent ROS generation within vSMC involves (i) Nox1-dependent transactivation of EGFR via c-Src; (ii) transactivation of EGFR leading to extracellular signal–regulated kinases (ERK1/2) phosphorylation; (iii) Nox1-dependent EGFR transactivation leading to MMP-9 activation via ERK1/2 phosphorylation, and (iv) Nox1/EGFR/MMP-9-induced N-cadherin shedding [71]. 

The mechanism by which Nox1 promotes migration via N-cadherin shedding and the role of N-cadherin in modulating SMC migration remain controversial, other, yet undefined, pathways may also be involved. It is unclear whether a diminished cofilin/MMP-9 axis contributes to the dysfunctional migratory response of Nox1-deficient cells in response to thrombin [71] or whether thrombin induced ROS engages a Slingshot-1L (SSH-1L)-cofilin pathway to regulate cytoskeletal organization and vSMC migration within vascular lesions. Nox1-mediated stimulation of MMP-9 may also be responsible for increased plasma cluster of differentiation-44 (CD44) levels in apoE^−/−^ mice following proteolytic cleavage of membrane-anchored CD44 [72]. While basic fibroblast growth factor (bFGF)- and PDGF-induced, Nox1-dependent migration of vSMC is dependent on c-Jun-N-terminal kinase (JNK) and Src/phosphoinositide-dependent kinase-1 (PDK1)/p21-activated protein kinase (PAK), respectively, thrombin-induced vSMC migration appears dependent on ROS-regulated p38 MAPK activation [73]. A Poldip2 mechanism for vSMC migration via regulation of focal adhesion turnover and traction force generation in a Nox4/RhoA/focal adhesion kinase (FAK)-dependent manner has also recently been reported [74]. While many of the aforementioned studies on Nox isoforms were performed using vSMC in culture, the likelihood that these cells are de-differentiated vSMCs [75] and/or stem cell derived myogenic progeny [18] and, thus, may not represent the putative response of differentiated vSMCs to changes in Nox activity cannot be ruled out. It remains to be seen whether these mechanisms observed in vitro are operational in vivo under physiological or pathophysiological conditions. Of note, previous studies have reported no changes in Src, p38 MAPK, or JNK in response to PDGF in wild-type or Nox1-deficient cells [69].

As arterial injury and arteriosclerotic lesions are characterized by enhanced thrombin expression and activity with increased Nox activity and ROS production [72], activation of this signalling cascade by thrombin identifies one potential mechanism by which Nox1-derived ROS may promote IMT.

Enhanced Nox-1 expression is also associated with elevated O_2_^−^ levels within intimal and medial vSMCs following carotid vascular injury [76]. Nox-derived ROS may also participate in neointimal formation by PDGF-induced signalling [77]. Indeed, ROS produced by Nox5 may play an important role in PDGF-induced JAK/Signal Transducer and Activator of Transcription (STAT) activation and vSMC proliferation [78]. PDGF is heavily dependent on JAK/STAT activation since specific knockdown of Nox5 reduces PDGF-induced ROS production and proliferation of these cell and inhibits PDGF-stimulated JAK2 and STAT3 phosphorylation [78]. Other studies have implicated Nox1 in vSMC phenotypic changes, including angiotensin II-induced hypertrophy [79], serum-induced proliferation [80], and bFGF-induced migration [81] and strain-induced phenotypic switching [82].Vascular SMC specific deletion of Nox1 reduced IMT in response to femoral artery wire injury [69] while ectopic expression of Nox1 exhibited increased PDGF-induced O_2_^−^ production in aortic vSMCs concomitant with increased growth and migration [69] . EGF, FGF-2, angiotensin II (AngII) and the plasminogen/plasmin system (uPA) all promote Nox activity with vSMC in vitro and contribute to IMT in vivo [69]. Finally, the precise mechanism of how Nox mediated ROS deteriorates vascular function and promotes vascular remodelling and IMT in vivo has been recently elucidated. Cyclophilin A (CyPA), a 20 kD chaperone protein that is secreted from vSMCs in response to ROS and stimulates vSMC proliferation and inflammatory cell migration in vitro and in vivo [79].

Despite the beneficial signalling role of Nox4 generated H_2_O_2_ from the endothelium, excessive concentrations from vSMC may induce inflammation, fibrosis, apoptosis, and even necrosis [83]. Given the constitutive nature of Nox4 activity, the regulation of H_2_O_2_ formation may be harmful or protective depending on the levels and source of ROS generated [60]. Under certain conditions, such as the release of transforming growth factor-β1, diabetes, and heart failure, Nox4-dependent H_2_O_2_ formation may become harmful. 

## 5. The Role of Nox in Adventitial Cells

**Figure 4:**
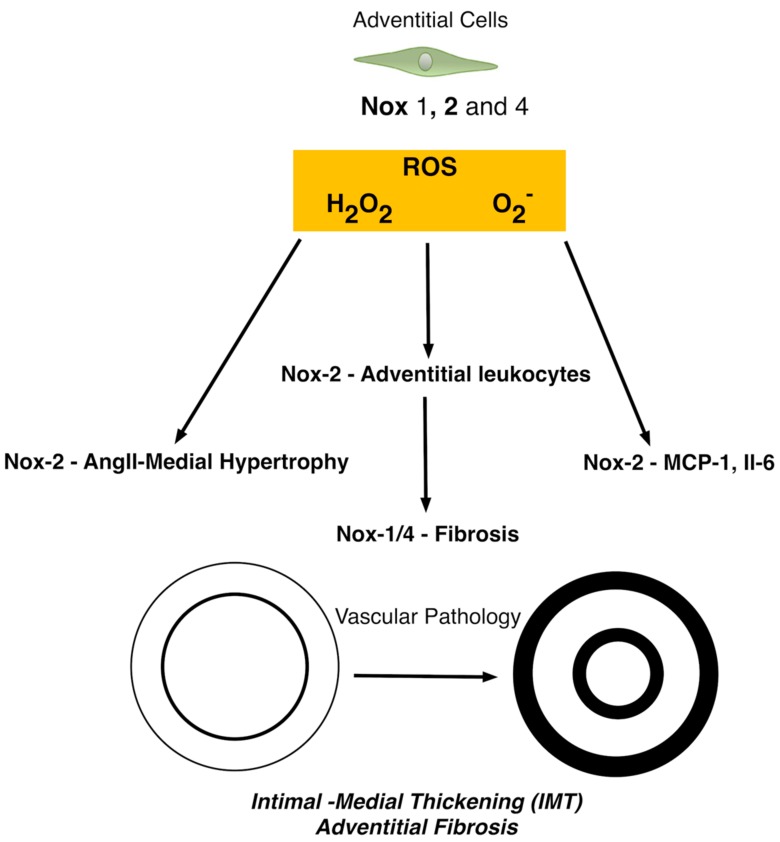
Simplified schematic of common forms of Nox enzymes in adventitial cells. Nox2 and p22phox are predominant in the adventitia of hypertensive vessels and promote the secretion of monocyte chemoattractant protein-1 (MCP-1) and interleukin 6 (IL-6). Nox1 and Nox4 with Nox4-derived H_2_O_2_ considered protective are also present [87].

The role of Nox isoforms within the adventitial layer has also been addressed (Figure 4). Arteriosclerotic vessels are characterized not only by IMT, but also increased stiffness secondary to collagen and elastin deposition, a process regulated by the adventitial fibroblast and termed fibrosis. The fibroblast is the primary cell type of the adventitial layer and contributes to the continual reorganization of the extracellular matrix via matrix deposition and secretion of growth factors, chemokines, and inflammatory cytokines [84]. Fibroblasts also influence and promote the inflammatory response by facilitating leukocyte recruitment, survival and function. The importance of the vascular adventitia to IMT and arteriosclerotic disease progression has also recently been highlighted [85]. Compelling evidence now suggests that the vascular adventitia is activated early following vascular injury and during IMT progression and may play an important role in vascular inflammation associated with arteriosclerotic disease [86]. Adventitial cells produce a large amount of Nox-derived ROS in response to vascular injury that can lead to an expanded vascularisation through the vasa vasorum and delivery of inflammatory cells to the adventitia and outer media culminating in vascular hypertrophy and hyperplasia [87].

Early studies revealed abundant co-localization of Nox2, with membrane p22phox and cytosolic p47phox and p67phox in aortic vascular adventitia and enrichment of p67phox-dependent Nox activity in cultured adventitial fibroblasts [88]. Adventitial cells also express Nox1 and Nox4 with Nox4-derived H_2_O_2_ considered protective. However, Nox2-derived ROS remains one of the main isoform for adventitial ROS signaling with adventitial leukocytes exhibiting significant expression [37]. Nox-2 is enhanced by several stimuli including hypoxia, cytokines, hormones, metabolic factors and mechanical injury [87].

Adventitial Nox2 generated ROS also plays an important role in angiotensin II-hypertension associated IMT by modulating the secretion of monocyte chemoattractant protein-1 (MCP-1) and interleukin 6 (IL-6) [89]. Adventitial Nox2 is activated in hypertension models [90] while ROS signaling also promotes vSMC hypertrophy via aquaporin 1 and Nox1 [91]. These studies suggest a putative role for adventitia-derived ROS in medial SMC hypertrophy and neointimal hyperplasia. The superoxide anion metabolite, H_2_O_2_ is the likely responsible for the adventitia-derived paracrine signalling across the vessel wall as it is highly stable, cell permeant, and capable of activating downstream signaling mechanisms in vSMCs, leading to phenotypic modulation and de-differentiation [86].

It is widely accepted that the fibroblast/myofibroblast is the cell most responsible for interstitial matrix accumulation and consequent structural deformations associated with adventitial fibrosis [92]. Accumulating evidence now suggests that oxidative stress resulting in ROS generation, mainly in the form of superoxide and hydrogen peroxide, plays a significant role in the initiation and progression of adventitial fibrosis [93]. Moreover, Nox4 mediated ROS-induced fibroblast cell activation is key event where it facilitates TGF-β1 signalling of fibroblast activation and differentiation into a profibrotic myofibroblast phenotype and matrix production [94].

## 6. The Role of Nox in Stem Cells

Resident stem/progenitor cells are characterized by their unique ability to self-renew and proliferate while retaining their ability to differentiate into specialized cells within the body. There are several reported resident vascular progenitor cells residing in all three layers of the vessel; the intima, media and adventitia, each displaying the ability to differentiate into vascular cell lineages [15]. These progenitor stem cell populations are multipotent and have the capability of differentiating into MSC-like cells [12]. Resident vascular MSCs-like cells are pericytes defined as multipotent stromal cells with the ability to differentiate into cells associated with mesodermal and neuroectodermal lineage including vSMCs, osteoblasts, adipocytes and chondrocytes [95].

ROS play an important role in dictating the fate of normal stem cells. Low levels of ROS are required for stem cells to maintain quiescence and self-renewal whereas increases in ROS production are associated with stem cell proliferation/differentiation, senescence, and apoptosis [96]. The production of ROS in stem cells is therefore by necessity tightly regulated to maintain tissue homeostasis and repair by various intrinsic and extrinsic factors, which may become altered leading to dysregulation of ROS production under various pathological conditions. The presence of Nox in stem cells is thought to have a functional role as O_2_ sensor and/or as a low-level ROS producer and redox messenger for controlling cell growth and differentiation [25]. 

In order to better understand the differentiation process, several studies have focused on the metabolic changes that occur during differentiation [97,98]. In an undifferentiated state, MSC reside in a special microenvironment called the stem cell niche. The purpose of this “niche” is to keep cells in an undifferentiated, quiescent state maintaining their stemness. For this to happen, O_2_ levels are kept at a minimum. When O_2_ levels are kept low, the stem cell niche is forced to turn to the use of an alternative anaerobic metabolism for energy [99,100]. This, in turn, results in low levels of ROS production due to the lack of available oxygen keeping proliferation activity at a minimum whilst maintaining the cells core stem cell properties of self-renewal and differentiation abilities. During the transition of stem cells from their undifferentiated state within the niche, several changes occur. The number of mitochondria present in the cell increases in response to O_2_ levels leading to increased levels of ROS production. Although this general process is present across all differentiation cells, MSCs display a unique redox pathway depending on their fate suggesting that MSCs have explicit redox profiles depending on their ultimate lineage specificity [101].

The main source of ROS in stem cells is the mitochondria. This oxidative reduction process is triggered by a leakage of a small number of electrons from the electron transport chain. These mitochondrial electrons react with molecular O_2_ resulting in formation of O_2_^−^, a precursor for ROS generation within the stem cell [102]. The oxidative reduction process is successfully achieved through the activity of several vital mitochondrial respiratory complex formations on the inner mitochondrial membrane space. The initial complex I formation is composed of Nox enzymes and acts as a catalyst during the oxidation of NADPH to NADP^+^ leading to the formation of superoxide radicals [102]. The main Nox enzymes in MSCs derived from adipose tissue responsible for this process are Nox1 and 4 [103]. Other sources of O_2_^−^ may be generated at varying levels in undifferentiated human MSCs by complex II comprising of succinate dehydrogenases [99]. The enzyme works by catalysing the oxidation of succinate to fumarate resulting in the conversion of flavin adenine dinucleotide (FAD) to its reduced form (FADH2) promoting ROS generation through intermediary electron transfer [99,102,104]. Complex III also known as co-enzyme q, bc1 complex is also associated with the generation of ROS in MSCs and is characterized by the presence of ubiquinol-cytochrome c reductase. Its main function is to catalyse the reduction of cytochrome c by the oxidation coenzyme Q. This oxidation process leads to electron leakage which in turn generates ROS [102,105]. The successful generation of ROS by complex III in MSCs via a cytochrome c reductase inhibitor (antimycin A) has also been reported [106]. As low levels of ROS provide a means for stem cell differentiation, this study highlights a possible approach to inducing the production of ROS in MSCs to initiate the differentiation process [106]. The presence of complex IV has been characterized; however, little is known about its role in ROS production in stem cells [102,107]. It is important to note that both complexes I and II have the ability to produce ROS within the mitochondrial matrix however complex II has the ability to produce ROS on both sides of the inner membrane facilitating easier access into the cytosol for signalling [108]. Although the mitochondria act as the main player in the generation of ROS in stem cells, it is not yet understood what is the specific role of each complex. Moreover, it is important to note that other organelle mechanisms of ROS generation may be important such as lysosomes, endoplasmic reticulum and the nuclear membrane [27]. 

**Figure 5:**
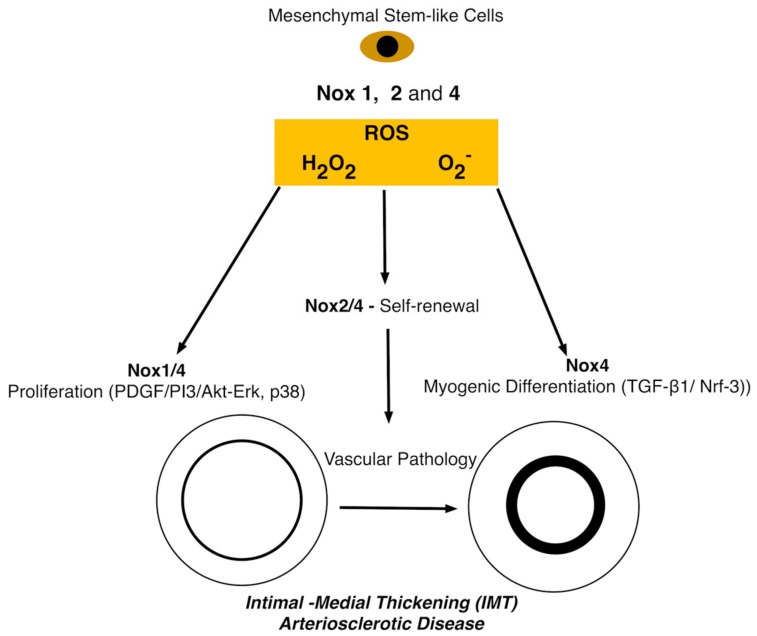
Simplified schematic of common forms of Nox enzymes in multipotent (mesenchymal stem cell (MSC)-like cells. Proliferative, self-renewing multipotent vascular progenitors with phenotypic characteristics of neural stem cells maintain a high ROS status and are highly responsive to ROS stimulation. ROS-mediated self-renewal and differentiation is dependent on PI3K/AKT signalling and underlies a redox-mediated regulatory mechanism of stem cell function and vascular repair [109].

In stem cells, the production of ROS by Nox enzymes is described as “controlled ROS generation”, carefully balanced by the presence of antioxidant/neutralization agents produced by the cellular antioxidant system thereby maintaining physiological properties (Figure 5) [109]. MSCs are said to have a balanced level of ROS production due to the presence of antioxidant regulators. Antioxidant regulators “scavenge” harmful oxidative products providing the cell with an oxidative defence system against oxidative stress and cellular apoptosis [96]. The main players involved in the vital neutralization of ROS in human MSCs are (*i*) SOD, driving the conversion of superoxide to O_2_ and H_2_O_2_ (*ii*) GPx enabling H_2_O_2_ reduction to H_2_O and (*iii*) catalase (CAT), responsible for the dismutation of H_2_O_2_ due to reaction with ferric states of heme-containing peroxidases [110].

The tight balance between the production of ROS and the activity of antioxidant players ensures homeostasis is maintained within the stem cell. Although antioxidants play an important role in maintenance of cellular homeostasis, it is important that production of ROS at low levels is permitted for activation of key pathways associated with stem cell proliferation, differentiation and survival. Low levels of ROS are associated with physiological properties of stem cells by their role in activating important signalling pathways involved in stem cell maintenance, proliferation and differentiation. There are many factors that induce low levels of ROS in MSCs including low levels of H_2_O_2_, hypoxia and the presence of inhibitors of the mitochondrial electron transfer chain e.g., rotenone and anti-mycin A [111]. 

Although the aforementioned factors influence the production of low levels of ROS, Nox enzymes themselves also have a role to play. The main Nox enzymes associated with the positive mediation of low levels of ROS in MSCs are Nox1, 2 and 4. Knockdown of Nox1 and 4 attenuated IL-17 dependent proliferation of hMSCs [112]. Similarly, Nox4 knockdown attenuated hypoxia-induced proliferation of adipose-derived stem cells (ASCs) [113]. Interestingly hypoxia induced Nox4 production of ROS stimulated the platelet derived growth factor receptor (PDGFR), AKT (PKB) protein kinases and the ERK signalling pathways whilst Nox4 knockdown showed significant downregulation indicating that activation of these pathways can induce ASC cell proliferation [113]. Similarly, the activation of Nox1 and 4 through growth factors and hypoxia resulting in upregulation of ROS has been shown to increase levels of MAPKs e.g., AKT, ERK and p38 promoting MSC proliferation [114]. Nox2 derived H_2_O_2_ regulates hippocampal stem/progenitor cells (AHPs) and intracellular growth signaling pathways to maintain their normal proliferation in vitro and in vivo [115]. Targeted silencing of Nox2/4 decreases intracellular ROS levels and total number of murine iPSCs resulting in reduced expression of insulin-like growth factor-1 (IGF-1), IGF-1 receptor and phosphorylation of ERK 1/2. Furthermore, expression of the stemness genes Sox2 and Oct4 was lower in Nox2/Nox4-deficient iPSCs, and higher in Nox2/Nox4-overexpressing iPSCs suggesting that Nox2-derived ROS contributes to stem cell pluripotency maintenance and self-renewal [116].

To date, several studies have shown a direct correlation between intracellular Nox-derived ROS and the modulation of multiple fates such as adipogenic, osteogenic and myogenic lineages. The importance of Nox4-dependent ROS generation during the early stages of adipocyte differentiation in rat bone-marrow derived MSCs has been postulated [117]. Of the seven isoforms of Nox enzymes, Nox4 was initially targeted due to the high expression levels of this isoform in pre-adipocytes. Similarly, using human adipose derived stem cells (ASCs) cultured under hypoxic conditions, cell proliferation increased concomitant with enhanced Nox-4 levels suggesting ROS plays a vital role in stimulating stem cell proliferation. Moreover, Nox4 knockdown attenuated this response [113]. Hypoxia induced generation of ROS mediated by Nox4 activation also increased PDGFR, AKT and ERK-related signalling pathways [118]. The benefit of Nox4-induced ROS seems to be a direct effect of its by-product, H_2_O_2,_ on the slow processes of adipogenic transition. Nox4 acts as a switch from insulin-induced proliferation to adipogenic differentiation by controlling MKP-1 expression which limits ERK1/2 signalling [119]. 

Multipotent neural stem cells (NSC) in contrast maintain high levels of ROS and are very responsive to ROS stimulation. ROS-mediated promotion of self-renewal and neurogenesis are dependent on PI3K/AKT (PKB) protein kinases signalling. Reducing cellular ROS levels interfered with normal NSC progenitor function both in vitro and in vivo [93]. As resident vascular stem cells are of neuroectoderm origin [12,120,121], this redox-mediated regulatory mechanism of stem cell function may have significant implications for vascular injury, disease, and repair.

### The Role of NADPH and the ROS Pathway during Vascular Myogenic Differentiation

The contributory role of resident MSC-like vascular progenitor stem cells to IMT and arteriosclerotic disease remains contentious [75]. Although myogenic differentiation of resident MSC-like cells can be initiated using biochemical factors such as TGF-β1, Notch signalling or PDGF in vitro, the micromechanical environment in which these resident vascular stem cells reside and their mitochondrial profile may ultimately dictate their myogenic differentiation capacity [122,123]. Indeed, biomechanical stimulation of stem cells is associated with ROS activation and regulation of Nox expression [124,125,126]. Although little is known about the mitochondrial profile and the role of NADPH during myogenic differentiation, several studies have been conducted on vascular stem cells, including MSC, ESCs and iPSCs. 

ROS can induce myogenic differentiation of stem cells into vSMCs [127], raising the possibility that ROS acts as a regulator of stem cell differentiation. However, little is known about how ROS is linked to myogenic differentiation. Recent studies support a role for sphingosylphosphorylcholine (SPC) in promoting SMC differentiation marker expression in human MSCs, an effect that was attenuated by treatment with a ROS inhibitor. Moreover, Nox-derived ROS affects redox-sensitive molecules including a multifunctional protein, protein deglycase (DJ-1) to drive myogenic differentiation in these cells [128]. The potential role of Nox4 mediated generation of H_2_O_2_ during ESC myogenic differentiation has also been assessed [127]. Sustained Nox4-H_2_O_2_ signalling resulted in enhanced ESC differentiation to vSMC after 4–12 days whilst Nox4 knockdown supressed myogenic differentiation [127]. Activation of Nox4 was mediated by the secretion of TGF-β1 from early differentiating ESCs. Nox4 then translocates from the cytoplasm to the nucleus leading to ROS production of H_2_O_2_ [127]. The Nox4 mediated H_2_O_2_ production causes phosphorylation of serum response factor (SRF) which co-localises to the nucleus and binds to the CArG on the promoter-enhancing region of vSMC specific genes [calponin1, myosin heavy chain 11, and transgelin] recruiting a muscle specific co-activator, myocardin [127]. Phosphatases are also potential targets of Nox4-derived ROS as such an association has been reported for Nox4 and protein-tyrosine-phosphatase 1 (PTP1) [129].

Similarly, ESC differentiation to vSMCs via Nox4 activity can be mediated by nuclear factor erythroid 2-related factor (Nrf) 3, a member of the cap “N” collar family of transcription factors. This is achieved through the translocation of Nrf from the endoplasmic reticulum to the nucleus activating SRF/myocardin complex formation as well as directly binding to the promoter region of vSMC specific genes [130,131]. Phospholipase A2, group 7 (Pla2g7) plays a crucial physiological role during this myogenic transition since a free radical scavenger and flavoprotein inhibitor of NADPH oxidase but not H_2_O_2_ inhibitor attenuated myogenic differentiation. Moreover, Nrf3 regulates (Pla2g7) gene expression through direct binding to the promoter regions of Pla2g7 gene [131]. 

Direct or indirect redox modulation of the PI3K/AKT and Wnt signaling pathways by Nox1 has also been reported and results in phosphorylation/inhibition of GSK3-β and β-catenin translocation into the nucleus as well as Notch1 activation in progenitor stem cells [132]. Loss of Nox1 results in increased phosphatase and tensin homolog (PTEN) activity that in turn inhibits AKT signalling pathway, as well as Wnt/β-catenin and Notch1 signalling. GSK3-β is an important mediator of Notch-Wnt crosstalk and, therefore, is a key player by which ROS regulates both pathways [132]. Nox2-derived ROS in bone-marrow (BM) also plays a critical role in mobilization, homing and the angiogenic capacity of endothelial progenitors (EPCs) and BM-derived stem/progenitor cells, thereby promoting revascularization of ischemic tissue [133].

While vSMCs are widely reported to undergo a phenotypic switch by de-differentiating and/or reprogramming to a synthetic phenotype during IMT and disease progression [9,11], resident vascular stem cell myogenic progeny from within the medial or adventitial layers may also play a significant role in IMT and arteriosclerotic disease [15]. Indeed, recent lineage tracing studies in mice have provided compelling evidence for the involvement of stem cell-derived progeny [12,19,134], in addition to “re-programmed” differentiated vSMC [135,136,137], as well as vSMC derived from EndoMT [14] in progressing IMT. These stem cells may become activated/re-programmed, differentiate down myogenic and myeloid lineages and subsequently dictate, in part, vessel remodelling. 

TGF-β1 is an important promoter of myogenic differentiation by regulating expression of key vSMC differentiation genes such as smooth muscle α-actin (SMA) and calponin1 (CNN1) through Nox4 derived ROS [138]. Indeed, Nox4 and myocardin-related transcription factor-A (MRTF-A), a transcription factor known to be important in expression of vSMC genes, are closely associated. MRTF-A interacts with the actin-binding protein, paladin [138] through phosphorylation of MRTF-A by a Rho kinase (ROCK). Nox4 knockdown decreases TGF-β-induced palladin expression and MRTF-A phosphorylation while palladin depletion decreases MRTF-A phosphorylation confirming a putative role for ROS regulation and suggesting Nox4-dependent palladin expression and phosphorylation of MRTF are critical factors in the regulation of SRF responsive gene expression in vSMCs [138]. Moreover, TGF-β1 generation of vSMC-like myofibroblasts as a key event in tissue fibrosis and vascular IMT progression has been associated with Hedgehog (Hh) responsive Gli adventitial stem cells [13,134]. TGF-β1 induced expression of Nox subunits and Hh signaling components is attenuated following Nox inhibition with ursolic acid, concomitant with reduced expression of α-SMA and type I collagen [139] suggesting a potential role for Nox in Hh promotion of IMT [140]. 

Recently, EndoMT, a recognised type of cellular transdifferentiation, has emerged as another possible source of tissue myofibroblasts contributing to IMT [14]. EndoMT is a complex biological process in which endothelial cells lose their specific markers and acquire a mesenchymal or myofibroblastic phenotype and express MSC-like markers including α smooth muscle actin (α-SMA) and type I collagen. Similar to epithelial mesenchymal transition (EMT), EndoMT can be induced by TGF-β1 [14]. Recent studies employing cell-lineage analysis have demonstrated that EndoMT may be important in the pathogenesis of pulmonary, cardiac, and vascular fibrosis, and may represent a novel therapeutic target for many fibrotic disorders [14]. Several mechanisms are involved in the profibrotic effects of TGF-β1, including Nox4 activation and transcriptional activation of numerous genes involved in the fibrotic process (collagens and proteoglycans, fibronectin) [141]. Nox2-mediated ROS production also promotes arterial EC specification in differentiating miPSCs by activating the Notch signaling pathway and contributes to the angiogenic potency of transplanted miPSC-derived EC [142]. 

Finally, within the arterial vasculature, mechanical and inflammatory redox signals, characteristic of vascular disease progression and IMT, have emerged as a secretagogues for bone morphogenic protein (BMP) production-with downstream activation of endothelial Nox expression. Paracrine signals provided by BMP and ROS augment aortic myofibroblast Msx2-Wnt signalling and matrix turnover leading to vascular calcification. Moreover, oxidation of vascular low-density lipoprotein (LDL) cholesterol generates oxysterols that trigger Runx2 activity via hedgehog signalling pathways [143]. Thus, BMP, Wnt, and hedgehog gene expression programs are elaborated within the vasculature via redox-regulated mechanisms underscoring oxidative stress as a major contributor to the pathobiology of arterial calcification.

## 7. Conclusions

Nox-derived ROS is pivotal to cellular signalling within the vascular compartment but can also cause dysfunction depending on spatiotemporal Nox expression. It is clear that expression and activity of Nox is tightly regulated, with both depleted and excessive Nox-derived ROS detrimental to the vasculature. Endothelial-specific overexpression of Nox clearly promotes vascular dysfunction and oxidative stress leading to disease progression. Similar effects are observed following overexpression of Nox in vSMCs and adventitial cells. At a low basal level, ROS plays a critical role in maintaining stem cell proliferation and survival of MSC-like cells. The putative role of Nox-derived ROS during myogenic differentiation of resident vascular MSC-like stem cells and/or adventitial fibroblasts has recently emerged and in this context, a greater focus on the Nox–derived ROS from a perivascular adventitial perspective is warranted to fully appreciate their contribution to disease progression. The development of Nox specific inhibitors has helped in understanding the role of each Nox isoform, and it is clear that a complex interplay among transcription factors, co-activators/-repressors and nuclear receptors, in addition to epigenetic mechanisms converge to cause Nox upregulation in several cardiovascular disorders [55]. Hence, a greater understanding of the mechanisms involved and revelation of the signalling molecules responsible for the increased expression and activation of Nox is imperative if these regulatory mechanisms are to be exploited therapeutically.
